# Metabolites of *Tilletia laevis* Suppress *Fusarium solani* Growth Through Metabolic Disruption and Oxidative Stress Responses

**DOI:** 10.3390/jof12090639

**Published:** 2026-08-26

**Authors:** Delai Chen, Shirong Ma, Weiwei Zhang, Dongbo Li, Yanni Chen, Wenqi Liu, Yali Wang, Xiaofei Yang, Liting Chen, Pin Liu, Duo Jin, Yan Ma, Jing Li, Muhammad Jabran

**Affiliations:** 1 College of Agriculture and Bioengineering, Longdong University, Qingyang 745000, China; lymasr@163.com (S.M.); tinglang2@163.com (D.L.); 18793836740@163.com (Y.C.); l3164251948@163.com (W.L.); 15569067290@163.com (Y.W.); estrella2320@163.com (X.Y.); 18093041397@163.com (L.C.); 18893442501@163.com (P.L.); 18272191526@163.com (D.J.); 17793097626@163.com (Y.M.); 18993458431@163.com (J.L.); 2 Gansu Key Laboratory of Protection and Utilization for Biological Resources and Ecological Restoration, Qingyang 745000, China; 3 College of Petroleum and Chemical Engineering, Longdong University, Qingyang 745000, China; nanmu0930@163.com; 4 College of Life Sciences, Shihezi University, Shihezi 832003, China; 5 State Key Laboratory for Biology of Plant Disease and Insect Pests, Institute of Plant Protection, Chinese Academy of Agricultural Sciences, Beijing 100193, China

**Keywords:** *Tilletia laevis*, *Fusarium solani*, fermentation broth, EC_50_, oxidative enzymes, biological control

## Abstract

*Fusarium solani* is a major soil-borne pathogen responsible for root rot diseases, posing a significant threat to agricultural productivity. The development of environmentally sustainable alternatives to chemical fungicides is therefore urgently needed. This study evaluated the antifungal activity of fermentation broths and extracellular metabolites derived from *Tilletia laevis* fungi at different developmental stages against *F. solani* under in vitro conditions. Six test agents, including carbendazim (one positive fungicide control), caffeic acid, phenylacetylglycine, and 6-hydroxypyridine-2-carboxylic acid (candidate bioactive metabolites), were evaluated for mycelial growth inhibition, EC_50_ values, and physiological responses. All treatments exhibited concentration-dependent inhibitory effects, with carbendazim showing the highest activity (EC_50_ = 21.26 mg L^−1^). Among the metabolites, 6-hydroxypyridine-2-carboxylic acid and phenylacetylglycine demonstrated notable antifungal efficacy, particularly at higher concentrations. Fermentation broths from the promycelial stage showed stronger inhibition than those from the teliospore stage, indicating stage-specific metabolite activity. Biochemical analyses of *F. solani* mycelia revealed significant changes in soluble protein, soluble sugar, and antioxidant enzyme activities (SOD and POD), suggesting that antifungal effects are mediated through metabolic disruption and oxidative stress. These findings highlight the potential of *T. laevis*-derived metabolites as eco-friendly biofungicides and provide a theoretical basis for sustainable management of soil-borne diseases. Further studies are required to identify active compounds and validate their efficacy under field conditions.

## 1. Introduction

*Tilletia laevis* Kühn, a basidiomycete fungus in the family Tilletiaceae, is the causal agent of common bunt (stinking smut) in wheat and represents an important soil-borne pathogen with complex developmental biology [1,2]. Its teliospores are characterized by thick, multilayered walls and distinctive morphological features, including a spherical-to-elliptical shape and grayish-brown coloration. These teliospores persist in soil and germinate through a series of well-defined developmental stages, namely teliospore activation, promycelial growth, primary basidiospore formation, H-body stage [3], and secondary basidiospore production, each associated with distinct metabolic profiles [4]. Increasing evidence suggests that these stage-specific metabolites may play critical roles not only in fungal development but also in interspecific microbial interactions.

*Fusarium solani*, a member of the Hyphomycetes, is a globally distributed soil-borne pathogen with a broad host range, infecting economically important crops across Solanaceae, Gramineae, and Leguminosae families [5]. It is a major cause of root and stem rot, leading to substantial agricultural losses. The pathogen exhibits strong survival in soil, persisting as chlamydospores, mycelia, or sclerotia for extended periods (up to 10 years), which complicates its management. Favorable environmental conditions, particularly moderate temperatures (~25 °C) and high humidity, further exacerbate disease incidence [6,7]. Previous work [8] has highlighted the increasing prevalence of *F. solani* in intensive agricultural systems and its emerging resistance to conventional fungicides, posing a significant challenge to sustainable crop protection.

Currently, the management of *Fusarium*-induced root rot relies heavily on chemical fungicides. Although effective in the short term, excessive and prolonged use of these chemicals has led to serious environmental contamination, adverse effects on human health [9], pesticide residues in food chains [10], and the development of resistant pathogen populations. In response, biological control [11,12] strategies have gained increasing attention as sustainable and eco-friendly alternatives. These approaches utilize beneficial microorganisms or their bioactive metabolites to suppress pathogen growth [13] and enhance plant health [14]. Notably, recent advances have demonstrated that microbial secondary metabolites [15,16], including phenolic compounds, organic acids, and nitrogen-containing heterocycles, can disrupt pathogen physiology by inducing oxidative stress, inhibiting cell wall synthesis, and altering metabolic pathways [17,18]. The antifungal activity observed in this study further supports the concept that fungal-derived metabolites can serve as effective bioactive compounds for agricultural applications [19,20]. Such metabolites have been shown to inhibit pathogen growth through multiple mechanisms, including disruption of membrane integrity, interference with nutrient uptake, and inhibition of enzymatic activity [21].

Despite these advances, significant research gaps remain. Most existing studies focus on well-known biocontrol agents such as *Bacillus* and *Pseudomonas* species, whereas comparatively little attention has been paid to the antifungal potential of metabolites derived from plant-pathogenic fungi such as *T. laevis*. Moreover, the stage-specific variation in metabolite composition during fungal development and its impact on antagonistic activity against other pathogens have not been systematically explored. In particular, the physiological and biochemical mechanisms underlying the interaction between *T. laevis* metabolites and *F. solani* remain poorly understood.

Therefore, the present study aims to evaluate the inhibitory effects of fermentation broths and extracellular metabolites of *T. laevis* at different developmental stages on the growth of *Fusarium solani*. Specifically, this study (i) quantifies antifungal activity using EC_50_ values, (ii) investigates changes in key physiological indicators such as soluble protein and soluble sugar content, and (iii) analyzes antioxidant enzyme responses, including superoxide dismutase (SOD) and peroxidase (POD) activities.

The novelty of this work lies in its integrative approach, combining antifungal bioassays with biochemical and enzymatic analyses to elucidate the mechanisms of pathogen inhibition. Furthermore, by focusing on stage-specific fungal metabolites, this study provides new insights into the ecological role of *T. laevis* and identifies potential candidates for developing novel, environmentally sustainable biofungicides. These findings contribute to advancing green disease management strategies and support the broader goal of sustainable agricultural production.

## 2. Materials and Methods

### 2.1. Experimental Materials

#### 2.1.1. Microbial Strains

*Tilletia laevis* (wheat smut fungus) and *Fusarium solani* were obtained from the College of Agriculture and Bioengineering, Longdong University. The *F. solani* strain was used as the target pathogen in all antifungal assays.

#### 2.1.2. Culture Media and Reagents

Potato dextrose agar (PDA) and soil leachate medium were used for fungal cultivation. Analytical-grade reagents included carbendazim, caffeic acid, phenylacetylglycine, and 6-hydroxypyridine-2-carboxylic acid. The selection of test agents was based on their reported antifungal or bioactive properties and their relevance for comparative analysis. Carbendazim was included as a commercial benzimidazole fungicide widely used as a reference standard in Fusarium control studies. Caffeic acid was selected as a naturally occurring phenolic compound with known antifungal and antioxidant activity. Phenylacetylglycine was included due to its reported involvement in microbial metabolic interactions and potential antimicrobial properties. 6-hydroxypyridine-2-carboxylic acid was selected as a nitrogen-containing heterocyclic compound with metal-chelating and antimicrobial potential. Fermentation broths from *Tilletia laevis* were included to evaluate stage-dependent biological activity. Therefore, the experimental panel was designed to compare a conventional fungicide reference with chemically distinct candidate metabolites in the same in vitro mycelial growth assay. Biochemical assays employed commercial kits for superoxide dismutase (SOD) and peroxidase (POD) activity (Beijing Box Biotechnology Co., Ltd., Beijing, China). Additional reagents included Coomassie Brilliant Blue G-250, 3,5-dinitrosalicylic acid (DNS), bovine serum albumin, and standard laboratory chemicals. Experiments were conducted using standard microbiological and biochemical equipment, including an autoclave, a laminar flow cabinet, an incubator, a centrifuge, a spectrophotometer, and an ultrasonic homogenizer.

### 2.2. Preparation of Culture Media and Pathogen Activation

PDA medium was prepared by boiling 200 g of peeled potato in 1 L of distilled water for 20–30 min, then filtering the mixture. Glucose (20 g) and agar (20 g) were added, and the volume was adjusted to 1 L. The medium was autoclaved at 121 °C for 20 min and poured into sterile Petri dishes under aseptic conditions. *Fusarium solani* cultures were revived from −20 °C storage, incubated at room temperature for 24 h, and subsequently cultured on PDA plates at 28 °C for 5 days, until active growth was observed, thereby activating and propagating the cultures [22]. Fermentation broths were used in crude form and expressed as g L^−1^ based on initial culture filtrate concentration. Pure compounds were tested at defined concentrations (mg L^−1^). No chemical extraction or dry mass normalisation was performed, and therefore, direct quantitative comparison between pure compounds and crude fermentation extracts should be interpreted with caution.

### 2.3. Cultivation of Tilletia laevis and Collection of Metabolites

Sterilized soil leachate medium without *Tilletia laevis* inoculation was used as the negative control for all fermentation broth experiments. The control was incubated, filtered, and processed in parallel with the treatment groups to ensure experimental consistency and to account for any potential antifungal effects of soil-derived organic and inorganic components or antibiotic residues. Soil leachate medium was prepared by extracting 75 g of soil with boiling distilled water and adjusting the volume to 1 L. The medium was sterilized at 121 °C for 20 min and cooled prior to use. Teliospores were surface-sterilized with 0.25% NaClO for 1 min, washed three times with sterile water, and inoculated into sterile soil leachate medium at a final concentration of 1 × 10^5^ spores mL^−1^. Antibiotics (penicillin and streptomycin) were added to prevent bacterial contamination. Cultures were incubated at 16 °C with shaking at 150 rpm. Extracellular metabolites were collected at two developmental stages: the teliospore activation stage (3 days) and the promycelial growth stage (5 days). Fermentation broths were centrifuged at 12,000 rpm (4 °C), and supernatants were collected, filtered, and stored at 4 °C for subsequent analysis. Sterilized soil leachate medium served as the control. For pure-compound assays, PDA plates containing the corresponding solvent or sterile-water background at the same final volume were used as negative controls. For fermentation-broth assays, sterilized soil leachate medium without *T. laevis*, supplemented with the same antibiotics and processed under the same incubation, centrifugation, filtration, and drying conditions, was used as the matched negative control. Inhibition percentage was calculated relative to the matched control for each treatment type.

### 2.4. In Vitro Antifungal Assay

The antifungal activity of test compounds was evaluated using the mycelial growth rate method. Test agents were prepared at three concentrations: chemical compounds at 20, 50, and 100 mg L^−1^, and fermentation broths at 20, 50, and 100 g L^−1^. Each treatment was incorporated into molten PDA (≈60 °C) and poured into Petri dishes. Plates without additives served as controls. Mycelial plugs (8 mm diameter) were excised from the actively growing margin of 5-day-old *F. solani* cultures grown on PDA. All plates were incubated at 28 °C for 6 days prior to measurement and sample collection. Colony diameter was measured using the cross-measurement method, and inhibition rate (%) was calculated as previously described by Vincent [23], Peccini et al. [24], and Araújo et al. [25].

$$\mathrm{Inhibition}\;\mathrm{rate}=\frac{\left(D_{c}\;-\;D_{t}\right)}{\left(D_{c}\;-\;D_{0}\right)}\times 100$$

where $D_{c}$ is control colony diameter, $D_{t}$ is the treatment diameter, and $D_{0}$ is the initial plug diameter. This approach aligns with standardized antifungal screening methods widely used in recent plant pathology studies.

### 2.5. Determination of Soluble Protein Content

Fungal mycelial biomass of *Fusarium solani* was harvested after exposure to the respective treatments, thoroughly washed with sterile distilled water to remove residual media, and homogenized under ice-cold conditions for subsequent enzyme extraction and biochemical analyses. Soluble protein content was quantified using the Coomassie Brilliant Blue G-250 method. A standard curve was prepared using bovine serum albumin (0–100 μg mL^−1^). Samples were mixed with dye reagent, incubated for 10 min, and then absorbance was measured at 620 nm using a spectrophotometer. Protein concentrations were calculated based on the regression equation derived from the standard curve. This method is consistent with current biochemical quantification approaches for fungal stress analysis. Standard calibration curves for soluble protein and soluble sugar determination were prepared as shown in Supplementary Tables S1 and S2, respectively. Representative standard curves are provided in Supplementary Figure S1.

### 2.6. Determination of Soluble Sugar Content

Biochemical analyses were performed using *F. solani* mycelial biomass collected from PDA plates after exposure to antifungal treatments. No conidial suspensions were used in the biochemical assays. Soluble sugars were determined using the DNS colorimetric method. Reaction mixtures containing glucose standards and DNS reagent were heated in a boiling water bath for 5 min, cooled, and diluted to 25 mL. Absorbance was measured at 540 nm, and sugar content was calculated using a standard curve. This method is widely used in recent studies to evaluate metabolic responses under stress conditions.

### 2.7. Determination of Antioxidant Enzyme Activities

#### 2.7.1. Preparation of Crude Enzyme Extract

Fungal mycelial biomass was harvested after treatment exposure, washed with sterile water, and homogenized under ice-cold conditions for enzyme extraction. Fungal samples were homogenized and sonicated on ice (200 W, 3 s pulses, 10 s intervals, repeated 30 times), followed by centrifugation at 8000× *g* for 10 min at 4 °C. The supernatant was collected as the crude enzyme extract.

#### 2.7.2. Superoxide Dismutase (SOD) Activity

SOD activity was determined using a commercial assay kit according to the manufacturer’s instructions. Absorbance was measured at 560 nm, and enzyme activity (U mL^−1^) was calculated based on inhibition of superoxide radical formation. The inhibition percentage was maintained between 30% and 70% to ensure measurement accuracy.

#### 2.7.3. Peroxidase (POD) Activity

POD activity was measured using the same extraction procedure as SOD and quantified using a commercial assay kit. Absorbance changes were monitored spectrophotometrically to reflect the hydrogen peroxide-scavenging capacity.

### 2.8. Statistical Analysis

All experiments were performed in triplicate. Data are expressed as mean ± standard deviation (SD). Differences were considered statistically significant at *p* < 0.05. EC_50_ values were estimated using linear regression analysis of log-transformed concentration versus inhibition response based on three concentration levels. Biochemical and enzyme-activity data were analysed separately for pure-compound and fermentation-broth experiments because the treatments were expressed in different concentration units. Goodness-of-fit was evaluated using R^2^ values. Standard curve preparation details are provided in Supplementary Materials. Toxicity regression equations and EC_50_ calculations for all treatments are provided in Supplementary Table S3.

## 3. Results

### 3.1. Antifungal Activity and Virulence Assessment

#### 3.1.1. Inhibitory Effects of Test Agents on *Fusarium solani*

**Figure 1:**
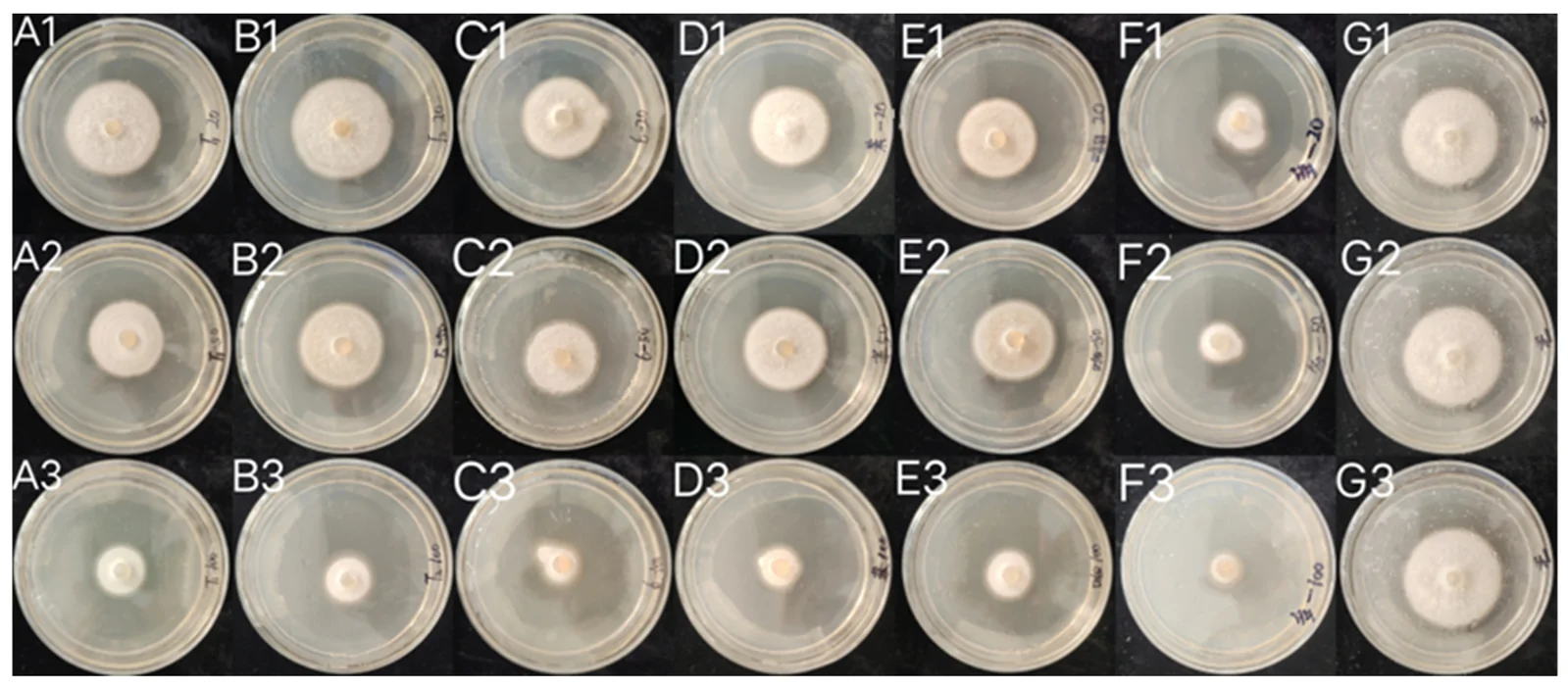
Effects of different antifungal agents on colony morphology and mycelial growth of *Fusarium solani*. Representative colony morphology of *F. solani* cultured on PDA medium supplemented with different antifungal agents: (**A**) activated teliospore fermentation broth of *Tilletia laevis*, (**B**) promycelial fermentation broth of *T. laevis*, (**C**) 6-hydroxypyridine-2-carboxylic acid, (**D**) phenylacetylglycine, (**E**) caffeic acid, (**F**) carbendazim, and (**G**) sterile water control. Images G1, G2, and G3 show the same sterile water control plate, reused for comparative illustration across different treatment concentrations. For each treatment, concentrations 1, 2, and 3 correspond to 20, 50, and 100 mg L^−1^ (or g L^−1^ for fermentation broths), respectively. Plates were incubated at 28 °C for 6 days before imaging. Increased inhibition of radial mycelial growth was observed with increasing treatment concentration.

The antifungal activity of six agents against *Fusarium solani* was evaluated based on colony diameter and inhibition rate. The selection of the six test agents was based on a structured comparative rationale to ensure coverage of chemically and biologically diverse antifungal candidates. As shown in Figure 1 and Table 1, all treatments exhibited inhibitory effects to varying degrees, with a clear concentration-dependent response. Among the tested agents, carbendazim consistently demonstrated the strongest inhibitory activity across all concentrations. Carbendazim was included as a standard positive control fungicide due to its well-established use as a systemic benzimidazole compound in plant disease management and its broad application against soil-borne *Fusarium* species in crops such as wheat, tomato, and cucumber [26]. It served as a benchmark for evaluating the relative antifungal efficacy and EC_50_ values of the tested compounds and fermentation-derived treatments under controlled in vitro conditions. The detailed toxicity regression parameters used for EC_50_ estimation are provided in Supplementary Table S3. At 20 mg L^−1^, carbendazim reduced colony diameter to 2.41 cm, with an inhibition rate of 51.78%; this increased to 73.58% at 100 mg L^−1^. In comparison, 6-hydroxypyridine-2-carboxylic acid and phenylacetylglycine showed moderate inhibition, particularly at higher concentrations (≥50 mg L^−1^), with inhibition rates exceeding 60% at 100 mg L^−1^. Caffeic acid exhibited relatively low inhibitory activity at lower concentrations but showed a substantial increase at 100 mg L^−1^ (50.80%). Fermentation broths from both developmental stages of *Tilletia laevis* displayed comparatively weaker antifungal effects, although inhibition increased with concentration. Notably, the promycelial fermentation broth exhibited higher inhibitory activity than the teliospore activation broth at equivalent concentrations.

#### 3.1.2. Concentration-Dependent Inhibition Patterns

**Figure 2:**
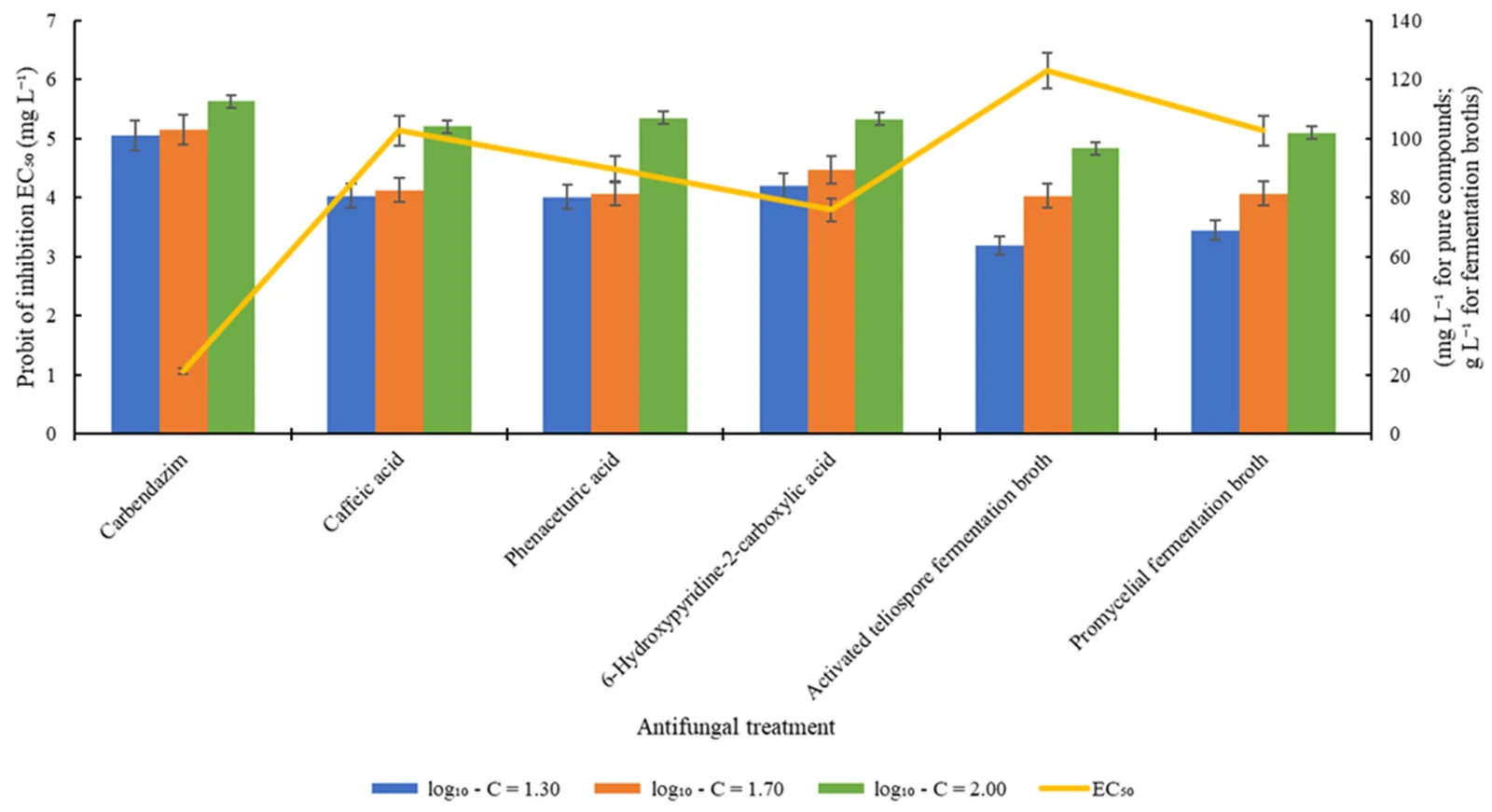
Probit-based toxicity responses and EC_50_ estimates of antifungal treatments against *Fusarium solani*. Bars represent mean probit-transformed inhibition responses at log_10_ concentrations of 1.30, 1.70, and 2.00, corresponding to treatment concentrations of 20, 50, and 100 mg L^−1^ for pure compounds and 20, 50, and 100 g L^−1^ for *Tilletia laevis* fermentation broths, respectively. Error bars represent the standard error of the mean (SE; *n* = 3). The line represents EC_50_ point estimates calculated from the corresponding toxicity regression equations. EC_50_ values are expressed in mg L^−1^ for pure compounds and g L^−1^ for fermentation-broth treatments. Sterile-water/solvent controls were used for pure compounds, whereas processed uninoculated soil-leachate medium was used for fermentation-broth treatments. The data show a descriptive comparison of antifungal activity and EC_50_ trends.

All treatments showed a positive correlation between concentration and inhibition rate, as illustrated in Figure 2. Carbendazim showed the steepest increase in inhibition, while fermentation broths demonstrated a more gradual response. EC_50_ values were estimated from regression analysis of three-point concentration response data and used for comparative assessment of antifungal potency. At all tested concentrations, the promycelial fermentation broth exhibited higher inhibitory activity than the teliospore-stage broth. For instance, at 100 g L^−1^, inhibition rates were 53.65% and 43.42%, respectively, indicating stage-specific differences in metabolite activity. Regression details are provided in Supplementary Table S3.

### 3.2. Effects on Soluble Protein and Soluble Sugar Contents

Biochemical assays were used on fungal mycelial biomass after treatment exposure. All tested pure compounds altered the soluble protein and soluble sugar contents of *F. solani*, although the responses varied among compounds and concentrations. Soluble protein content was lower in all compound-treated groups than in the sterile-water control. 6-hydroxypyridine-2-carboxylic acid produced the greatest reductions, with protein content remaining close to zero across the tested concentrations. Carbendazim also strongly reduced soluble protein content, whereas caffeic acid produced a less pronounced and non-monotonic response, with comparatively higher protein levels at 20 and 100 mg L^−1^.

**Figure 3:**
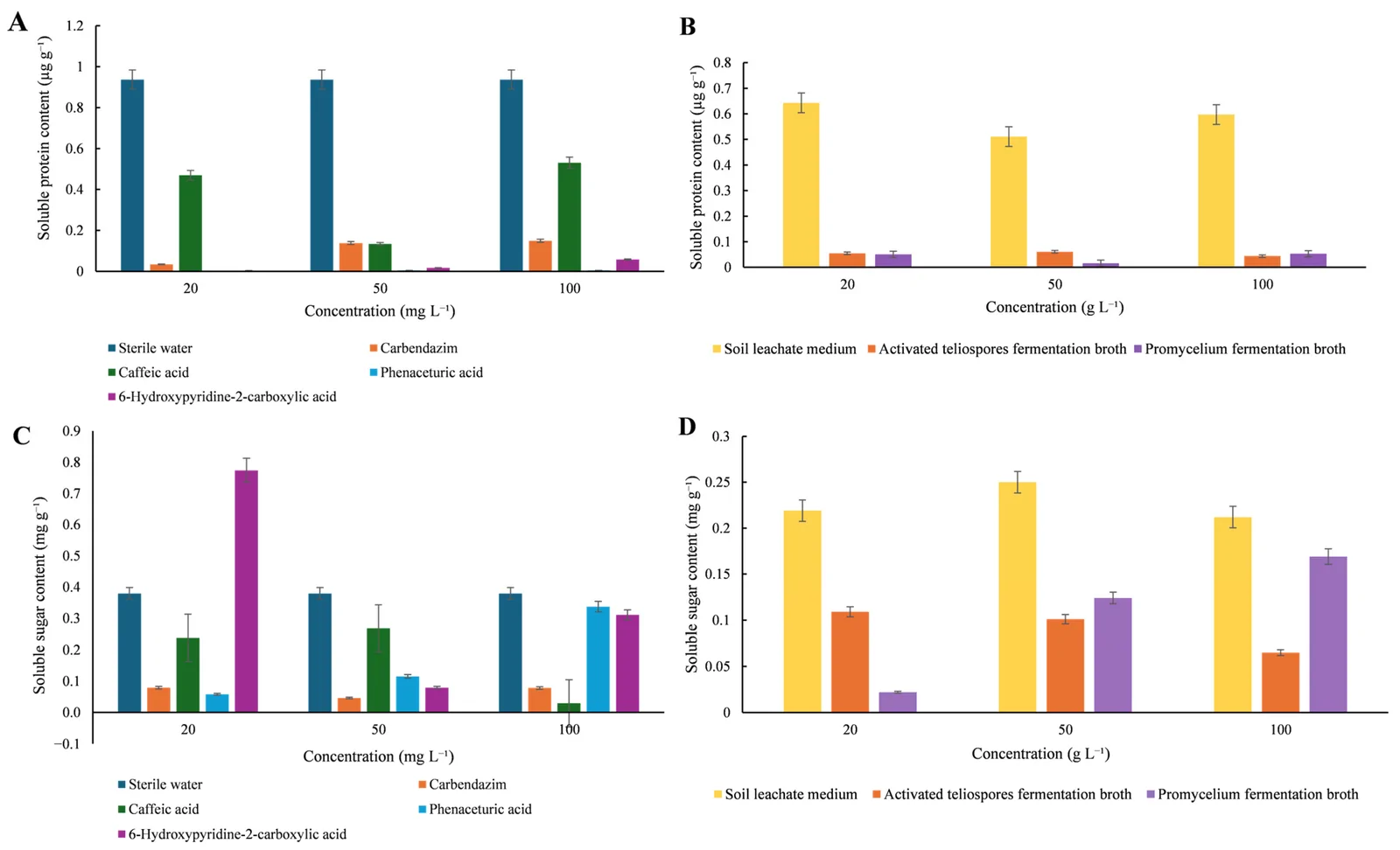
Effects of pure antifungal compounds and *Tilletia laevis* fermentation broths on soluble protein and soluble sugar contents in *Fusarium solani*. (**A**) Soluble protein content following treatment with carbendazim, caffeic acid, phenylacetylglycine and 6-hydroxypyridine-2-carboxylic acid at 20, 50 and 100 mg L^−1^; sterile water was used as the negative control. (**B**) Soluble protein content following treatment with activated-teliospore and promycelial fermentation broths at 20, 50 and 100 g L^−1^; uninoculated soil-leachate medium processed under the same conditions was used as the matched negative control. (**C**) Soluble sugar content following treatment with the pure compounds. (**D**) Soluble sugar content following treatment with the fermentation broths. Bars represent mean ± standard deviation of three independent biological replicates (*n* = 3). Data are presented as descriptive comparisons of treatment effects.

Both *T. laevis* fermentation broths markedly reduced soluble protein content compared with the uninoculated soil-leachate control at all tested concentrations. The activated-teliospore broth maintained consistently low protein levels, while the promycelial broth produced the lowest protein content at 50 g L^−1^. Soluble sugar content also remained lower than the matched soil-leachate control. The activated-teliospore broth showed a gradual decline in sugar content with increasing concentration, whereas the promycelial broth showed an increasing trend from 20 to 100 g L^−1^. Overall, the fermentation broths caused strong but stage-specific alterations in the protein and carbohydrate metabolism of *F. solani*. Full datasets for soluble protein and soluble sugar contents are presented in Supplementary Table S4 and Figure 3.

### 3.3. Antioxidant Enzyme Activity

SOD activity was consistently high in carbendazim and phenylacetylglycine treatments across concentrations. In contrast, caffeic acid and 6-hydroxypyridine-2-carboxylic acid showed increased SOD activity primarily at higher concentrations. POD activity exhibited distinct trends depending on treatment. Carbendazim and 6-hydroxypyridine-2-carboxylic acid showed increasing POD activity with concentration, whereas phenylacetylglycine and teliospore fermentation extracts showed decreasing trends. Antioxidant enzyme activities varied among treatments and concentrations, indicating induction of oxidative stress responses and disruption of cellular redox homeostasis in *F. solani*.

**Figure 4:**
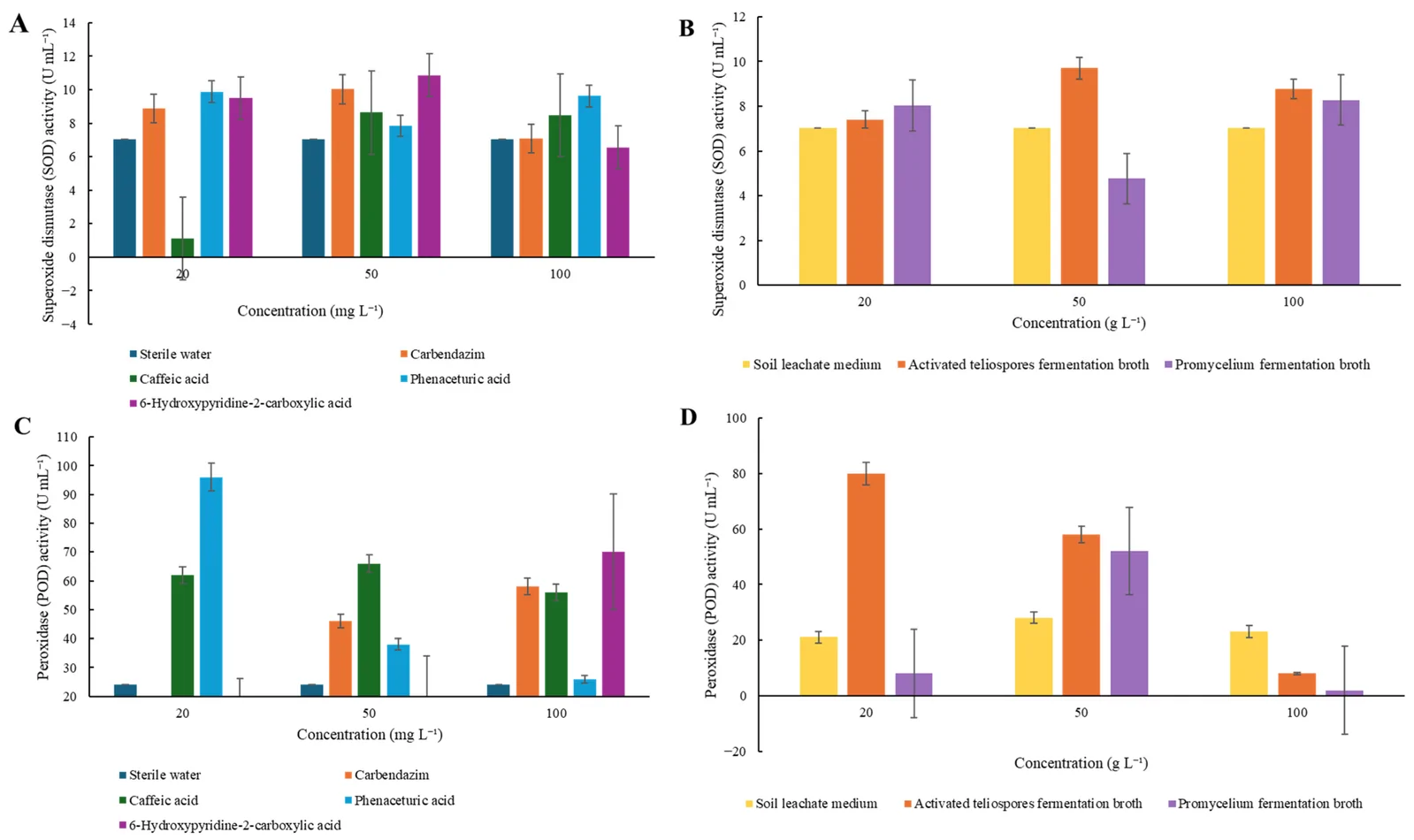
Effects of pure antifungal compounds and *Tilletia laevis* fermentation broths on antioxidant enzyme activities in *Fusarium solani*. (**A**) Superoxide dismutase activity following treatment with carbendazim, caffeic acid, phenylacetylglycine and 6-hydroxypyridine-2-carboxylic acid at 20, 50 and 100 mg L^−1^; sterile water was used as the negative control. (**B**) Superoxide dismutase activity following treatment with activated-teliospore and promycelial fermentation broths at 20, 50 and 100 g L^−1^; uninoculated soil-leachate medium processed under the same conditions was used as the matched negative control. (**C**) Peroxidase activity following treatment with the pure compounds. (**D**) Peroxidase activity following treatment with the fermentation broths. Bars represent mean ± standard deviation of three independent biological replicates (*n* = 3). Data are presented as descriptive comparisons of treatment effects.

In the fermentation-broth experiment, SOD activity in the soil-leachate control remained comparatively stable across concentrations. The activated-teliospore broth increased SOD activity, with the strongest response at 50 g L^−1^. The promycelial broth increased SOD activity at 20 and 100 g L^−1^ but produced a pronounced reduction at 50 g L^−1^. The activated-teliospore fermentation broth induced high POD activity at 20 and 50 g L^−1^, followed by a substantial decline at 100 g L^−1^. The promycelial broth produced low POD activity at 20 g L^−1^, a marked increase at 50 g L^−1^ and very low activity at 100 g L^−1^. These contrasting SOD and POD responses suggest that the fermentation broths affected cellular redox regulation in a developmental-stage- and concentration-dependent manner. Thus, the two developmental-stage broths induced contrasting antioxidant responses. Complete data for antioxidant enzyme activities (SOD and POD) are provided in Figure 4 and Supplementary Table S5.

## 4. Discussion

*Fusarium solani* is a highly adaptable soil-borne pathogen with strong persistence in agricultural ecosystems [27], making it a major constraint to sustainable crop production. Its ability to survive in soil for extended periods and rapidly colonize host tissues contributes to the difficulty of effective disease management [28]. Environmental factors such as soil type, temperature, and moisture have also been shown to significantly influence the development and severity of Fusarium root rot diseases [7]. These factors may also affect the efficacy of microbial metabolites, highlighting the importance of evaluating biocontrol agents under diverse environmental conditions. In addition, microbial metabolites such as Bacillus-derived lipopeptide biosurfactants have been reported to inhibit *F. solani*, supporting the broader relevance of metabolite-based antifungal strategies [29]. Conventional control strategies rely heavily on chemical fungicides; however, increasing concerns regarding environmental contamination, pesticide residues, and the emergence of resistant pathogen populations have significantly reduced their long-term efficacy. A previous study [30] have highlighted the urgent need for alternative strategies, particularly those based on microbial metabolites and environmentally benign bioactive compounds.

In this study, all tested agents exhibited inhibitory activity against *F. solani*, with a clear concentration-dependent response. Carbendazim was included as a positive control due to its well-documented use as a systemic benzimidazole fungicide for Fusarium species, serving as a benchmark for evaluating the relative antifungal efficacy of the tested compounds. Carbendazim demonstrated the highest antifungal efficacy, consistent with previous reports describing its broad-spectrum activity through inhibition of β-tubulin assembly and disruption of fungal mitosis. However, the relatively strong inhibitory effects observed for 6-hydroxypyridine-2-carboxylic acid and phenylacetylglycine at higher concentrations suggest that these compounds may serve as promising alternatives or complementary agents in biocontrol strategies. These findings are consistent with previous reports demonstrating the effectiveness of microbial biocontrol agents against *Fusarium solani*. For example, actinomycetes-derived metabolites have been shown to inhibit pathogen growth and reduce disease severity significantly [31]. Similarly, endophytic fungi and rhizospheric bacteria suppress Fusarium infections by producing bioactive compounds and inducing plant defense responses [22,32]. Previous studies have demonstrated that soil-borne actinomycetes exhibit strong antagonistic activity against *Fusarium solani*, significantly reducing disease incidence in chickpea [31]. Similarly, endophytic fungi have been reported to provide eco-friendly management of root rot diseases by enhancing plant defense responses and inhibiting pathogen colonization [22].

In addition, bacterial biocontrol agents such as Bacillus velezensis and Pseudomonas fluorescens have shown promising antifungal activity through mechanisms including antibiotic production, nutrient competition, and induction of systemic resistance [32,33]. Forest-associated and marine-derived microorganisms have also demonstrated inhibitory effects against *Fusarium solani*, highlighting the ecological diversity of potential biocontrol agents [34,35,36]. The differential antifungal activity observed between fermentation broths derived from the teliospore activation and promycelial growth stages of *Tilletia laevis* indicates that metabolite composition varies significantly across developmental stages. The higher inhibitory activity of promycelial-stage extracts suggests that bioactive secondary metabolites accumulate during this stage, thereby enhancing antagonistic interactions. Fungal biocontrol agents, particularly Trichoderma harzianum, have been shown to suppress Fusarium-induced diseases through mycoparasitism, enzyme secretion, and secondary metabolite production [37]. However, most studies have focused on well-known microbial agents, while metabolites derived from plant-pathogenic fungi such as *Tilletia laevis* remain largely unexplored.

Biochemical analyses further revealed significant alterations in soluble protein and sugar content in *F. solani* following treatment. The increase in soluble protein content under caffeic acid treatment, particularly at higher concentrations, suggests the induction of stress-responsive protein synthesis, possibly involving heat shock proteins or detoxification enzymes. In contrast, the elevated soluble sugar content observed under 6-hydroxypyridine-2-carboxylic acid treatment indicates an adaptive osmotic response, as soluble sugars are known to function as osmoprotectants under stress conditions. These findings are consistent with recent reports [38,39,40] demonstrating that fungal pathogens modulate carbohydrate metabolism and protein expression to cope with antifungal stress.

The observed biochemical and enzymatic changes suggest that antifungal treatments induce metabolic disruption and oxidative stress in *Fusarium solani*, leading to growth inhibition. The observed changes in antioxidant enzyme activities provide further insight into the underlying mechanisms of antifungal action. Superoxide dismutase (SOD) and peroxidase (POD) are key components of the cellular defense system against oxidative stress. In this study, increased SOD activity in carbendazim and phenylacetylglycine treatments suggests enhanced scavenging of superoxide radicals, while the concentration-dependent increase in POD activity under carbendazim and 6-hydroxypyridine-2-carboxylic acid treatments indicates elevated hydrogen peroxide detoxification. These patterns suggest that the tested compounds induce the accumulation of reactive oxygen species (ROS), thereby triggering oxidative stress in *F. solani* cells. These results agree with previous studies indicating that biocontrol agents induce oxidative stress in fungal pathogens, leading to cellular damage and growth inhibition [37,41]. Additionally, microbial metabolites have been reported to disrupt membrane integrity and metabolic pathways, further contributing to pathogen suppression [34].

Recent studies have also demonstrated that beneficial microorganisms such as Streptomyces can enhance plant resistance to Fusarium infections by coordinating rhizosphere microbial communities and activating plant defense-related gene expression [42]. This suggests that microbial metabolites not only act directly on pathogens but may also contribute to systemic resistance mechanisms in host plants. The observed biochemical responses may also be linked to complex chemical interactions in the rhizosphere, where microbial metabolites act as signaling molecules influencing pathogen behavior and plant defense responses [43]. These findings are consistent with recent studies demonstrating that microbial metabolites regulate gene expression, stress responses, and metabolic pathways in both plants and pathogens [44]. The contrasting decrease in POD activity observed in treatments with teliospore extracts and phenylacetylglycine at higher concentrations may indicate enzyme inhibition or unrepaired cellular damage, further supporting the role of oxidative stress in antifungal activity.

Although the results demonstrate the promising antifungal activity of *T. laevis*-derived metabolites, several limitations should be acknowledged. The lack of consistent statistical significance for some parameters may be due to experimental variability, including potential contamination, fluctuations in metabolite stability, or differences among reagent batches. Moreover, the study was limited to in vitro assays and did not include plant-level or field validation, which is essential for confirming practical efficacy. Additionally, only two developmental stages of *T. laevis* were investigated, and the specific chemical composition of the active metabolites was not characterized. Recent studies have also demonstrated that integrated biocontrol strategies targeting multiple pathogens, including fungi and nematodes, can enhance plant growth and disease resistance under greenhouse conditions [45]. Similarly, marine-derived actinomycetes have shown strong potential for controlling Fusarium diseases in tomato, both in vitro and in vivo [35], highlighting the broader applicability of microbial metabolites in sustainable agriculture.

However, this study is limited to in vitro assays and does not include ultrastructural or metabolomic validation. In addition, a limitation of this study is that fermentation-derived treatments were evaluated in crude form without dry mass normalization. EC_50_ values were derived from a limited number of concentration points (*n* = 3) and, therefore, represent preliminary estimates. Future studies should include expanded concentration ranges (5–6 points) to generate full sigmoidal dose–response curves and more accurately determine EC_50_. Therefore, comparisons between pure compounds and fermentation broths reflect relative biological activity rather than absolute potency based on equivalent bioactive mass. Future research should focus on identifying and characterizing the active compounds responsible for antifungal activity using advanced analytical techniques such as LC-MS/MS and metabolomics. Furthermore, in planta experiments and field trials are necessary to validate the effectiveness of these metabolites under natural conditions. Integrating molecular approaches, such as gene expression analysis of oxidative stress pathways and cell wall synthesis genes, would provide deeper insights into the mechanisms of pathogen inhibition. Overall, this study highlights the potential of *T. laevis* fermentation metabolites as a source of novel bioactive compounds for sustainable disease management. By targeting multiple physiological pathways, including growth inhibition, metabolic disruption, and oxidative stress induction, these metabolites offer a promising basis for developing an environmentally friendly biofungicide candidate.

## 5. Conclusions

This study demonstrates that *Tilletia laevis* fermentation metabolites exhibit inhibitory activity against *Fusarium solani*, with effectiveness dependent on compound type and developmental stage. While carbendazim showed the highest antifungal activity, metabolites such as 6-hydroxypyridine-2-carboxylic acid and phenylacetylglycine displayed promising bioactivity, particularly at higher concentrations. Enhanced inhibition by promycelial-stage extracts highlights the importance of stage-specific metabolite production. Biochemical responses, including changes in soluble protein, soluble sugar, and antioxidant enzyme activities, indicate that antifungal effects are associated with metabolic disruption and oxidative stress. These findings suggest that *T. laevis*-derived metabolites act through multi-target mechanisms. Although limited to in vitro conditions, this study provides a foundation for developing a potential eco-friendly biofungicide candidate. Future research should focus on metabolite identification, elucidation of molecular mechanisms, and validation under greenhouse and field conditions.

## Figures and Tables

**Table 1:** In vitro antifungal activity of six test agents and fermentation broths derived from *Tilletia laevis* against *Fusarium solani*.

Treatment	Concentration	Colony Diameter (cm)	Inhibition Rate (%)	Log Concentration	*p* Value
Sterile water (control)	-	5.00 ± 0.37	-	-	-
Carbendazim	20 mg L^−1^	2.41 ± 0.18	51.78 ± 0.50	1.30 ± 0.31	5.05 ± 0.42
	50 mg L^−1^	2.21 ± 0.05	55.78 ± 0.17	1.70 ± 0.09	5.15 ± 0.18
	100 mg L^−1^	1.32 ± 0.14	73.58 ± 0.25	2.00 ± 0.15	5.63 ± 0.22
Caffeic acid	20 mg L^−1^	4.17 ± 0.17	16.68 ± 0.33	1.30 ± 0.06	4.03 ± 0.24
	50 mg L^−1^	4.04 ± 0.09	20.33 ± 0.15	1.70 ± 0.31	4.13 ± 0.17
	100 mg L^−1^	2.11 ± 0.28	50.80 ± 0.07	2.00 ± 0.10	5.20 ± 0.15
Phenylacetylglycine	20 mg L^−1^	4.19 ± 0.16	16.20 ± 0.24	1.30 ± 0.04	4.01 ± 0.24
	50 mg L^−1^	4.11 ± 0.64	17.76 ± 0.13	1.70 ± 0.53	4.07 ± 0.17
	100 mg L^−1^	1.82 ± 0.21	63.67 ± 0.18	2.00 ± 0.29	5.35 ± 0.09
6-Hydroxypyridine-2-carboxylic acid	20 mg L^−1^	3.95 ± 0.50	21.00 ± 0.25	1.30 ± 0.64	4.20 ± 0.38
	50 mg L^−1^	3.51 ± 0.31	29.80 ± 0.34	1.70 ± 0.33	4.47 ± 0.12
	100 mg L^−1^	1.85 ± 0.08	62.92 ± 0.27	2.00 ± 0.07	5.33 ± 0.06
Activated teliospore fermentation broth	20 g L^−1^	4.83 ± 0.22	3.50 ± 0.33	1.30 ± 0.14	3.19 ± 0.35
	50 g L^−1^	4.18 ± 0.37	15.00 ± 0.17	1.70 ± 0.57	4.03 ± 0.02
	100 g L^−1^	2.85 ± 0.34	43.42 ± 0.12	2.00 ± 0.29	4.83 ± 0.27
Promycelial fermentation broth	20 g L^−1^	4.69 ± 0.85	6.14 ± 0.11	1.30 ± 0.83	3.45 ± 0.15
	50 g L^−1^	4.14 ± 0.16	17.34 ± 0.37	1.70 ± 0.17	4.07 ± 0.19
	100 g L^−1^	2.31 ± 0.19	53.65 ± 0.16	2.00 ± 0.35	5.10 ± 0.28

Note: Values are expressed as mean ± standard deviation (SD) of three biological replicates. *p* values were derived from regression-based toxicity analysis, and log-transformed concentration values were used to estimate EC_50_. Inhibition values were calculated relative to their respective matched controls. Pure compounds were compared with sterile water/solvent controls, while fermentation-derived treatments were compared with sterilized water controls.

## Data Availability

The original data presented in this study are available within the article. Additional data supporting the findings of this study are available from the corresponding author upon reasonable request.

## Supplementary Materials

The following supporting information can be downloaded at: https://www.mdpi.com/article/10.3390/jof12090639/s1, Table S1. Preparation scheme for bovine serum albumin standards used for soluble protein determination. Table S2. Preparation scheme for glucose standards used for soluble sugar determination. Table S3. Toxicity regression equations, coefficients of determination (R^2^), and EC_50_ values of six agents against *Fusarium solani*. Table S4. Effects of different agents on soluble protein and soluble sugar contents in *Fusarium solani*. Table S5. Effects of different agents on superoxide dismutase (SOD) and peroxidase (POD) activities in *Fusarium solani*. Figure S1. Standard curves used for biochemical quantification assays. (A) A bovine serum albumin (BSA) standard curve was generated for the determination of soluble protein content using the Coomassie Brilliant Blue G-250 method. (B) A glucose standard curve was generated for the determination of soluble sugar content using the 3,5-dinitrosalicylic acid (DNS) colorimetric assay. Regression equations were used to calculate protein and sugar concentrations in *Fusarium solani* samples following antifungal treatments.
