# Supplementary material for: Metabolites of Tilletia laevis Suppress Fusarium solani Growth Through Metabolic Disruption and Oxidative Stress Responses
Source: J Fungi (Basel). 2026 Aug 26;12(9):639. doi: 10.3390/jof12090639 (PMC13608498; doi:10.3390/jof12090639)
Supplement: Supplementary file 1 [file jof-12-00639-s001.zip › jof-4355840-supplementary.pdf]

Supplementary material

Table S1. Preparation scheme for bovine serum albumin standards used for soluble protein determination.

Tube no.	Bovine serum albumin (mL)	Protein content (μg)	Distilled water (mL)	Coomassie Brilliant Blue G-250 (mL)
0	0.0	0	1.0	3.0
1	0.2	20	0.8	3.0
2	0.4	40	0.6	3.0
3	0.6	60	0.4	3.0
4	0.8	80	0.2	3.0
5	1.0	100	0.0	3.0

Note: BSA standards ranging from 0 to 100 μg mL^{-1} were prepared for the generation of the standard calibration curve using the Coomassie Brilliant Blue G-250 method. Absorbance was measured at 620 nm, and protein concentrations of fungal samples were calculated based on the regression equation derived from the standard curve.

Table S2. Preparation scheme for glucose standards used for soluble sugar determination.

Tube no.	1 mg mL^{-1} glucose standard solution (mL)	Distilled water (mL)	DNS reagent (mL)	Glucose content (mg)
0	0.0	2.0	1.5	0.0
1	0.2	1.8	1.5	0.2
2	0.4	1.6	1.5	0.4
3	0.6	1.4	1.5	0.6
4	0.8	1.2	1.5	0.8
5	1.0	1.0	1.5	1.0

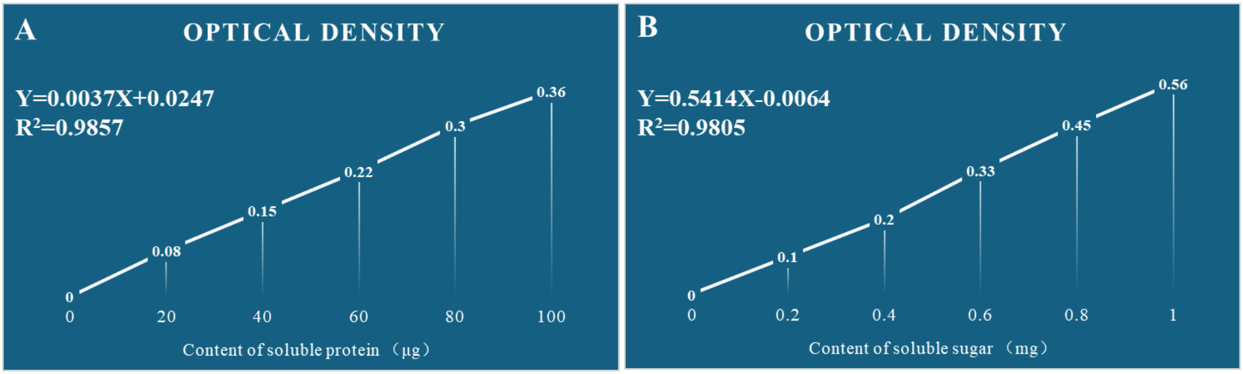

Figure S1. Standard curves used for biochemical quantification assays. (A) A bovine serum albumin (BSA) standard curve was generated for the determination of soluble protein content using the Coomassie Brilliant Blue G-250 method. (B) A glucose standard curve was generated for the determination of soluble sugar content using the 3,5-dinitrosalicylic acid (DNS) colorimetric assay. Regression equations were used to calculate protein and sugar concentrations in *Fusarium solani* samples following antifungal treatments.

Table S3. Toxicity regression equations, coefficients of determination (R^2), and EC_{50} values of six agents against *Fusarium solani*.

Treatment	Toxicity regression equation	R^2	EC_{50}
Carbendazim	y = 0.8077x + 3.9278	0.8170	21.26 mg L^{-1}
Caffeic acid	y = 1.5887x + 1.8044	0.7423	102.68 mg L^{-1}
Phenylacetylglycine	y = 1.8202x + 1.4455	0.7140	89.70 mg L^{-1}
6-Hydroxypyridine-2-carboxylic acid	y = 1.5760x + 2.0375	0.8705	75.82 mg L^{-1}
Activated teliospore fermentation broth	y = 2.3310x + 0.1284	0.9956	123.00 g L^{-1}
Promycelial fermentation broth	y = 2.3063x + 0.3610	0.9498	102.66 g L^{-1}

Note: EC_{50} values were calculated using regression analysis of log-transformed concentrations versus inhibition probability values. Concentrations of pure compounds (carbendazim, caffeic acid, phenylacetylglycine, and 6-hydroxypyridine-2-carboxylic acid) are expressed in mg L^{-1}. In contrast, fermentation broth treatments (activated teliospore fermentation broth and promycelial fermentation broth) are expressed in g L^{-1}. Higher R^2 values indicate better goodness of fit for the regression model. Lower EC_{50} values indicate stronger antifungal activity. EC_{50} values were estimated from three concentration levels and should be interpreted as comparative indicators rather than absolute pharmacological constants.

Table S4. Effects of different agents on soluble protein and soluble sugar contents in *Fusarium solani*.

Treatment	Concentration	Soluble protein (μg g^{-1})	Soluble sugar (mg g^{-1})
Sterile water (control)	0	0.937 ± 0.15	0.379 ± 0.64
Carbendazim	20 mg L^{-1}	0.034 ± 0.13	0.079 ± 0.51
	50 mg L^{-1}	0.138 ± 0.07	0.046 ± 0.09
	100 mg L^{-1}	0.149 ± 0.51	0.078 ± 0.25
Caffeic acid	20 mg L^{-1}	0.469 ± 0.19	0.238 ± 0.31
	50 mg L^{-1}	0.134 ± 0.03	0.268 ± 0.08
	100 mg L^{-1}	0.531 ± 0.14	0.029 ± 0.72
Phenaceturic acid	20 mg L^{-1}	0.003 ± 0.38	0.058 ± 0.11
	50 mg L^{-1}	0.004 ± 0.25	0.115 ± 0.18
	100 mg L^{-1}	0.004 ± 0.50	0.337 ± 0.14
6-Hydroxypyridine-2-carboxylic acid	20 mg L^{-1}	0.003 ± 0.17	0.774 ± 0.26
	50 mg L^{-1}	0.017 ± 0.15	0.079 ± 0.38
	100 mg L^{-1}	0.057 ± 0.31	0.311 ± 0.41
Activated teliospore fermentation broth	20 g L^{-1}	0.055 ± 0.19	0.109 ± 0.39
	50 g L^{-1}	0.061 ± 0.02	0.101 ± 0.16
	100 g L^{-1}	0.044 ± 0.14	0.065 ± 0.07
Promycelial extract	20 g L^{-1}	0.051 ± 0.22	0.022 ± 0.19
	50 g L^{-1}	0.016 ± 0.36	0.124 ± 0.11
	100 g L^{-1}	0.053 ± 0.24	0.169 ± 0.27

Note: Soluble protein content was determined using the Coomassie Brilliant Blue G-250 assay, while soluble sugar content was measured using the DNS colorimetric method. Values are presented as mean ± SD from three independent biological

replicates. Changes in protein and sugar content reflect metabolic responses of *F. solani* to antifungal stress.

Table S5. Effects of different agents on superoxide dismutase (SOD) and peroxidase (POD) activities in *Fusarium solani*.

Treatment	Concentration	SOD activity ($U\ mL^{-1}$)	POD activity ($U\ mL^{-1}$)
Sterile water (control)	0	7.02 ± 0.08	24.35 ± 0.37
Carbendazim	20 mg L^{-1}	8.89 ± 0.37	8.11 ± 0.18
	50 mg L^{-1}	10.03 ± 0.25	46.03 ± 0.26
	100 mg L^{-1}	7.08 ± 0.17	58.09 ± 0.11
Caffeic acid	20 mg L^{-1}	1.11 ± 0.34	62.13 ± 0.08
	50 mg L^{-1}	8.64 ± 0.62	66.02 ± 0.19
	100 mg L^{-1}	8.47 ± 0.33	56.16 ± 0.34
Phenaceturic acid	20 mg L^{-1}	9.88 ± 0.29	96.27 ± 0.53
	50 mg L^{-1}	7.84 ± 0.14	38.36 ± 0.19
	100 mg L^{-1}	9.63 ± 0.52	26.29 ± 0.22
6-Hydroxypyridine-2-carboxylic acid	20 mg L^{-1}	9.50 ± 0.24	6.44 ± 0.08
	50 mg L^{-1}	10.87 ± 0.37	14.39 ± 0.14
	100 mg L^{-1}	6.56 ± 0.21	70.04 ± 0.35
Activated teliospore fermentation broth	20 g L^{-1}	7.41 ± 0.08	80.42 ± 0.26
	50 g L^{-1}	9.70 ± 0.24	58.28 ± 0.17
	100 g L^{-1}	8.76 ± 0.04	8.43 ± 0.14
Promycelial extract	20 g L^{-1}	8.03 ± 0.16	8.29 ± 0.27
	50 g L^{-1}	4.76 ± 0.37	52.28 ± 0.35
	100 g L^{-1}	8.28 ± 0.46	2.36 ± 0.11

Note: Superoxide dismutase (SOD) and peroxidase (POD) activities were measured using commercial enzyme assay kits following exposure to different antifungal treatments. Values are expressed as mean ± SD of three biological replicates. Variations in enzyme activities indicate oxidative stress responses induced by antifungal compounds and fermentation metabolites.
